# Entropy-Weight-Method-Based Integrated Models for Short-Term Intersection Traffic Flow Prediction

**DOI:** 10.3390/e24070849

**Published:** 2022-06-21

**Authors:** Wenrui Qu, Jinhong Li, Wenting Song, Xiaoran Li, Yue Zhao, Hanlin Dong, Yanfei Wang, Qun Zhao, Yi Qi

**Affiliations:** 1 School of Mathematics and Statistics, Qilu University of Technology (Shandong Academy of Sciences), University Road 3501, Changqing District, Jinan 250353, China; qwr@qlu.edu.cn (W.Q.); lijinhong@qlu.edu.cn (J.L.); 201892010067@stu.qlu.edu.cn (W.S.); 10431210873@stu.qlu.edu.cn (X.L.); 201911010029@stu.qlu.edu.cn (Y.Z.); 201911110033@stu.qlu.edu.cn (H.D.); 201804300168@stu.qlu.edu.cn (Y.W.); 2 Department of Transportation Studies, Texas Southern University, 3100 Cleburne Street, Houston, TX 77004-9986, USA; qun.zhao@tsu.edu

**Keywords:** entropy weight method, traffic flow forecasting, k-nearest neighbors algorithm, neural network

## Abstract

Three different types of entropy weight methods (EWMs), i.e., EWM-A, EWM-B, and EWM-C, have been used by previous studies for integrating prediction models. These three methods use very different ideas on determining the weights of individual models for integration. To evaluate the performances of these three EWMs, this study applied them to developing integrated short-term traffic flow prediction models for signalized intersections. At first, two individual models, i.e., a k-nearest neighbors (KNN)-algorithm-based model and a neural-network-based model (Elman), were developed as individual models to be integrated using EWMs. These two models were selected because they have been widely used for traffic flow prediction and have been approved to be able to achieve good performance. After that, three integrated models were developed by using the three different types of EWMs. The performances of the three integrated models, as well as the individual KNN and Elman models, were compared. We found that the traffic flow predicted with the EWM-C model is the most accurate prediction for most of the days. Based on the model evaluation results, the advantages of using the EWM-C method were deliberated and the problems with the EWM-A and EWM-B methods were also discussed.

## 1. Introduction

The entropy weights method (EWM) is a commonly used information-weighting method in decision making. It has been widely used in comprehensive evaluation studies that use different evaluation indexes [1,2,3]. In these studies, the weights of different indexes are determined according to the degree of dispersion. The smaller the entropy value, the greater the degree of dispersion of the index, and the greater the influence of the index on the comprehensive evaluation. Therefore, it should be signed with a greater weight [2]. Recently, EWM has been used in integrating different prediction models to get better predictions [4,5,6]. In these studies, the weights of different models, which quantitatively measure the importance of each model, were determined based on the degree of dispersion of the prediction errors. However, there are two different opinions on determining the weight of an individual model. Some studies believe that a smaller information entropy value means that the data are provided by many useful attributes, so a larger weight should be assigned and vice versa [4,7]. On the contrary, some studies suggest that a smaller entropy value of the prediction error indicates that the variation degree and uncertainty of model prediction is greater, and thereby, a smaller weight should be assigned to this model and vice versa [5,8,9,10]. One recent study [6] indicates that there is a nonlinear relationship between the entropy value and model accuracy level. Both low-accuracy and high-accuracy prediction models can result in small entropy values of the model prediction errors. Thus, the weight cannot be assigned based on the entropy value alone. To address this problem, they proposed a new entropy weight method for model integration. The prediction accuracy level of each individual model was incorporated into the calculated weights to reduce the impact of the model with less accuracy, which results in the improved prediction accuracy of the integrated model.

These three different EWMs have all been used by researchers for integrating prediction models [4,5,6,7]. They use very different ideas on determining the weights of individual models for integration. However, there is a lack of research that compares the performance of these different methods and identifies the best EWM for integrating prediction models. To address this problem, in this research, these three different entropy-based methods were applied to develop an integrated model to predict the short-term traffic at signalized intersections. Their performances were compared and analyzed based on the results of this study.

Short-term traffic flow prediction is crucial for advanced traffic management, especially for complex urban roadway networks. The main challenge in studying traffic flow problems is that the traffic flow data are unevenly distributed, highly dimensional, and dynamic changing [11]. Entropy analysis has been applied to traffic and transportation planning since the 1980s [12,13]. Previous studies applied entropy-based methods to identify different levels of the orderliness of traffic flow in a roadway network for the purposes of incident detection, roadway safety analysis, and driving behavior analysis [11,14,15,16,17,18].

In this study, at first, two individual traffic flow prediction models, i.e., a k-nearest neighbors (KNN)-algorithm-based model and a neural-network-based model (Elman), were developed because these two types of models have been widely used for traffic flow prediction and have been approved to be able to achieve good performance [19,20,21,22,23]. After that, three integrated models were developed by using the three different entropy-based methods. The developed models were evaluated by comparing the predicted traffic flow rates with the traffic data collected at a real-world signalized intersection. Finally, the model performance was analyzed, and conclusions and recommendations of this study were provided.

## 2. Literature Review

### 2.1. Entropy Weight Method (EWM)

The EWM is one of the weighting methods that measures the dispersion level of different information sources in decision making. It has been widely used in comprehensive evaluation studies where the weights of different indexes are determined according to the entropy value of the different evaluation indexes. For example, Dang and Dang [2] used a multi-standard decision-making method to evaluate the environmental quality of the Organization for Economic Cooperation and Development countries. The weights and method standards were determined based on the entropy weight method. Zhao et al. [1] developed an entropy-based model to predict automobile engine fault diagnosis. The weight of each factor in the evaluation was determined based on entropy. In all these comprehensive evaluation studies, a smaller entropy value of the indicator means a greater degree of dispersion, thereby it has a greater impact and should be assigned a greater weight.

Except for comprehensive evaluation studies, researchers also applied EWM methods for integrating different prediction models to improve prediction accuracy. In these studies, the weights of different models were determined based on the entropy of the model prediction errors. There are two different opinions on determining the weight of each individual model. Some studies believe that a model with a smaller entropy value of prediction error should be assigned a greater weight. For example, in a study [4], to predict the critical frequency of the ionosphere, authors used the entropy method to assign weights to the two single prediction results of Union Radio Scientifique Internationale and the International Radio Consultative Committee to develop an integrated prediction model. In this study, it was stated that a small information entropy value means the data are provided by many useful attributes, so a large weight should be assigned to this model. In another study [7], to increase the prediction accuracy of software reliability failure data, authors established an interacted prediction model using the EWM. In this study, it was believed that if the value of information entropy is smaller, the uncertainty is smaller, and greater weight should be given. It can be concluded that for the above papers, the basic idea for assigning weights to different models is that the smaller the entropy value of the prediction error of an individual model, the greater the weight should be assigned. This type of EWM is referred to as type A EWM (EWM-A) in this study.

On the contrary, some other studies believe that a smaller entropy value of the prediction error indicates that the variation degree and uncertainty of model prediction is greater, thereby a smaller weight should be assigned to this model. For example, to accurately predict the Normalized Vegetation Difference Index (NDVI) in the Yellow River basin, Huang et al. [5] developed a forecasting model by combining three individual models, i.e., multilinear regression (MLR), artificial neural network (ANN), and support vector machine (SVM) models. The method used to determine the weight is EWM. The idea is that if the prediction error of a single prediction model varies greatly, the entropy value of the model is small, indicating that the model does not perform well and should be given a small weight. In another study, Sun et al. [8] used the same EWM to assign weights to the gray GM (1,1) model and the gray Verhulst model for predicting the bearing capacity of anchor bolts. Chen and Li [9] also used the same EWM to develop an integrated prediction model for unit crop yield prediction. To predict sintering energy consumption, Wang et al. used this EWM to assign weights to two sintering energy consumption models [10]. For all the above papers that used EWM for model integration, the basic idea for assigning weights to different models is that if an individual model has a smaller entropy value of prediction error, the prediction variance in a model is larger, and a smaller weight should be assigned to it. This type of EWM is referred to as type B EWM (EWM-B) in this study.

Besides these two commonly used EWMs, recently, Shan and Zhang [6] proposed another EWM-based method for model integration. The authors indicate that there is a nonlinear relationship between the entropy value and model accuracy level. Both low-accuracy and high-accuracy prediction models can result in small entropy values of the model prediction errors. Thus, the weight cannot be assigned based on the entropy value alone. To address this problem, they proposed using a weighted entropy of the model prediction error, and the prediction accuracy level of the individual model was incorporated into this weighted entropy. In this way, the impact of the model with low accuracy can be reduced and the integrated model can be improved. This type of EWM is referred to as type C EWM (EWM-C) in this study.

In this paper, three integrated traffic flow prediction models were developed by using these three different types of EWMs, introduced above. Regarding the individual models, traffic flow forecasting has been intensively studied. Both parametric and non-parametric models were developed. Among all these models, the K-Nearest Neighbor (KNN) Algorithm and Artificial Neural Network (ANN) were approved to have good performance in predicting short-term traffic flow [19,20,21,22,23]. Following is a brief introduction to the literature that used these two methods for developing traffic flow prediction models.

### 2.2. Short-Term Traffic Flow Forecasting—K-Nearest Neighbor (KNN) Algorithm

The K-Nearest Neighbor Algorithm (KNN), a classic non-parametric regression method, has been widely used in short-term traffic forecasting. It has been approved to be able to achieve good performance [24,25,26,27,28]. In these studies, several KNN-based models were developed by improving the basic KNN algorithm. In summary, the KNN algorithm can be improved in four aspects:

1. Extended state vector.
  State vector describes the criterion by which the current data are compared with historical data. Usually, the state vector *X*(*t*) is defined as *X*(*t*) = [*S*(*t*), *S*(*t* − 1), …, *S*(*t* − *n*)], where *S*(*t*), *S*(*t* − 1), …, *S*(*t* − *n*) denote the traffic flow rates at time intervals *t*, *t* − 1, …, *t* − *n*, respectively. Some research [25,26,27,28] added spatial factors (such as the upstream and downstream intersection traffic flow rates) to extend the dimension of the state vector.
2. Improved distance measurements.
  The common method of measuring “proximity” in non-parametric regression is to use Euclidean distance [29,30] or weighted Euclidean distance [29] to calculate the distance between state vectors. There are other distance measuring methods that have been utilized by researchers, such as Manhattan distance [29,30,31,32], Hassanat distance [33,34], and Chi-square [35]. Improvements include using the weighted Euclidean distance by considering different factors. For example, Yu et al. suggested that weights should be assigned based on the close degree between time components in the state vector and the forecasting time [26]. Habtemichael and Cetin also recommended giving more weight to the recent measurements and less to the older ones [36].
3. Improved methods for determining the *K* value.
  Based on the calculated distance, the *K* nearest neighbors can be identified. The KNN model is sensitive to the selected *K* value, and the *K* value affects the model accuracy [37]. Previous studies have used different methods to determine the *K* value based on average absolute percentage error, relative error, and root mean square error [24,25,26,27,28,38,39,40].
4. Enhanced prediction algorithm.
  For the KNN method, the model prediction is mainly based on the simple average or weighted average of the K nearest neighbors. There are different methods to determine the weights. For example, ref. [25,26,27] used the inverse distance as the weight, and ref. [28] used the Gaussian function to determine the weights of the selected neighbors.

### 2.3. Short-Term Traffic Flow Forecasting—Artificial Neural Network (ANN)

Artificial neural network (ANN) is another widely used forecasting method. It has non-linear mapping and non-parametric characteristics and has great application potential in traffic flow prediction [41]. Many researchers have applied the ANN or Back Propagation (BP) neural network to predict traffic flow rate or congestion levels [27,42,43,44,45,46,47,48,49,50,51]. Recently, a dynamic feedback neural network called Elman was used in traffic flow prediction and showed improved results [19,20,21,22,23]. Elman neural network adds a context layer to the network, which makes the output of the network at the current moment not only depend on the current inputs but also related to the inputs at the previous moment using a memory function. This feature makes the Elman model outperform the traditional BP model [21].

## 3. Methodology

### 3.1. Data Description

We selected a signalized intersection in China to collect the data used for the model training and validation. Traffic information from 1 October 2018 to 1 April 2019, at this intersection, was collected, for a total of 156 days. The collected traffic data include:

- Traffic flow rates by signal cycle;
- Queue length;
- Signal timing plan;
- Weekend or not.

At this study intersection, traffic signal cycle lengths are 1.5 min in some periods and 2 min in others; therefore, traffic flow data are aggregated at 6 min intervals, and the traffic flow rate here is the vehicles arriving at the intersection every 6 min. The data were separated into two groups, the training group and the validation group. The validation group contains six days of traffic data (27 March–1 April) and the training group includes the rest of the data. The data were also grouped into weekday data and weekend data. Since the traffic patterns are different during the week, one prediction model was developed for each day of the week. Note that there are no data available on Tuesday to develop a prediction model due to system maintenance.

### 3.2. Model Development

At first, two individual models were developed, one KNN-based and one ANN-based model. In our previous study [21], three individual models, i.e., basic KNN, BP, and Elman models were developed. The model evaluation results showed that the Elman model outperformed the BP model. Thus, the Elman model was selected as an ANN-based model for model integration in this study. A detailed introduction of this model can be found in our previous published paper [21]. In addition, in this study, we improved the basic KNN model developed by Qu et al. [21] in three different ways, including using weighted distance measurement, optimizing the *K* value, and improving the prediction algorithm. The KNN model developed in this study is referred to as the improved KNN model. After developing the two individual models, i.e., Elman model and the improved KNN model, the three different EWM methods that we introduced before will be used for integrating these two individual models. Finally, the results of different types of integrated models will be compared to identify the best EWM method for integrating different prediction models. In the following sections, the development of the improved KNN algorithm and three EWM-based integrated models will be introduced first.

#### 3.2.1. Improved K-Nearest Neighbor’s Algorithm

In Qu et al. [21], a basic KNN model was developed. In this research, an improved KNN model was developed by using weighted distance measurement, optimizing the *K* value, and improving the prediction algorithm.

1. Weighted Distance Measurement:
  The model developed in this research is to forecast the vehicle arrival rate at the intersection 30 min later based on the arriving rates in the previous 3 h. Therefore, the prediction model can be mathematically expressed as follows.
  (1) $$f\left(x_{t-29,\;}\ldots x_{t-1},\;x_{t}\;\right)=x_{t+5}{}_{}$$
  where,
  t is the current time interval;
  $x_{t}$ is the arrival traffic flow rate during the current time interval.
  Since the traffic flow rate is at a 6 min interval, the vector ($\;x_{t-29},\;\ldots \;x_{t-1},x_{t}$) represents the arrival travel flow rates during the previous 3 h and $\;x_{t+5}$ represents the predicted traffic flow rate that will arrive at the intersection in half an hour. According to Habtemichael and Cetin [36], the time factor should be considered in the traffic flow prediction, which means when calculating the similarity between current and historical traffic flow data, more weight should be given to the more recently collected traffic flow data. According to this idea, the following weighted Euclidean distance is used:
  (2) $$d_{ij}=\sqrt{{\sum_{t=T-29}^{T}\omega_{t}\times \left(x_{it}-y_{jt}\right)}^{2}}$$
  (3) $$\omega_{t}=\frac{W_{t,norm}}{\sum_{t=T-29}^{T}W_{t,norm}}$$
  where,
  $x_{it}$ is the number of vehicles arriving at the *t*th time interval on the *i*th day in the historical dataset;
  $x_{jt}\;$is the number of vehicles arriving at the *t*th time interval on the *j*th day in the prediction dataset;
  $\omega_{t}$ is a time-related weight coefficient;
  $W_{t,\;norm}$ is the normalized temporal distance between the endpoint of *t*th time interval and the prediction time point, which can be expressed as follows:
  (4) $$W_{t,norm}=\frac{W_{t}-W_{\min}}{W_{\max}-W_{\min}}$$
  where,
  $W_{t}$ is the temporal distance between the endpoint of *t*th time interval and the prediction time point (in the number of time intervals as the unit);
  $W_{max}$ is the longest temporal distance from the prediction time point;
  $W_{min}$ is the shortest temporal distance from the prediction time point.
2. Optimized *K* Value
  Based on the distance calculated in Equation (2), the *K* nearest neighbors (the *K* historical days that have the traffic conditions most similar to the traffic condition at the targeted time *t* of the prediction day) can be selected. In the basic KNN model that was developed by Qu et al. [21], a given *k* value (*K* = 10) was used. To improve the model prediction, in this study, different *K* values from 7 to 15 were tested and the *K* values that resulted in the lowest prediction error were selected for predicting the traffic flow rate at the study intersection.
3. Improved Prediction Algorithm
  In the basic KNN model developed by Qu et al. [21], the average traffic flow rate of the selected *K* days was used for prediction. In this study, the weighted average method is used and the neighboring distance is used as the weight. The basic idea is that if the traffic condition of the selected day is more similar to the predicted day, it should contribute more to the predicted traffic flow rates. Thus, the weighting coefficient of each neighbor can be calculated by Equation (5).
  (5) $$w_{i}=\frac{1/d_{ij}}{\sum 1/d_{ij}}$$
  where $d_{ij}$ represents the weighted Euclidean distance between the *i*th similar historical day and the prediction day (*j*th day) and is calculated by using Equation (2). Then, the predicted traffic flow at the given time *t* + 5 can be estimated using Equation (6).
  (6) $${\hat{x}}_{t+5}=\sum_{i=1}^{k}w_{i}x_{i(t+5)}{}^{*},$$
  where $x_{i\left(t+5\right)}^{*}$ represents the number of vehicles arriving 30 min after the target time *t* during the *i*th historical day that was one of the selected *K* nearest neighbors.

#### 3.2.2. Integrated Prediction Models Based on Entropy Weight Method

The three different EWM methods that we introduced before will be used for integrating the two individual models, i.e., improved KNN and Elman models. Following are the introductions of these three EWMs.

1. Entropy Weight Method A (EWM-A)
  As mentioned in the literature review section, the EWM-A method is based on the idea that the smaller the entropy value of the prediction error of an individual model, the greater the weight should be assigned to it and vice versa. According to Bai et al. (2020), by using the EWM-A method, the two selected individual models can be integrated through the following process:
  Step 1: Calculate the absolute error weight of the individual model at time *t* by Equation (7).
  (7) $$p_{st}=\frac{\left|e_{st}\right|}{\sum_{t=1}^{m}\left|e_{st}\right|}\;\;(s=1,2,\cdots ,n;t=1,2,\cdots ,m)$$
  where,
  $e_{st}=\left|{\hat{y}}_{st}-y_{t}\right|$,
  *s* indicates different models,
  *n* is the number of individual models (*n* = 2 in this study),
  *t* represents the time, *m* is the number of prediction time points,
  ${\hat{y}}_{st}$ is the predicted value of the *s*th individual model at time *t*,
  $y_{t}$ is the observed value.
  Step 2: Calculate the entropy value of the *s*th individual model:
  (8) $$H_{s}=-k\sum_{t=1}^{m}p_{st}\ln p_{st}\;\;(s=1,2,\cdots ,n)$$
  If $P_{st}=0$, then $P_{st}\ln P_{st}=0$, $k=\frac{1}{\ln m}$
  Note that, according to the entropy concept, $P_{st}$ in Equation (8) should be a probability of an event. However, according to Equation (7), $P_{st}$ is a ratio of a prediction error to the sum of prediction errors instead of a probability. This is a critical problem with this type of EWM and will be discussed more in the model evaluation part.
  Step 3: Calculate the weight of the *s*th individual model:
  (9) $$\omega_{s}=\frac{1-H_{s}}{n-\sum_{s=1}^{n}H_{s}}\;\;(s=1,2,\cdots ,n)$$
  In this study *n* = 2, thus, $\omega_{s}$ becomes:
  (10) $$\omega_{s}=\left\{\begin{array}{cc} \frac{1-H_{1}}{2-H_{1}-H_{2}} & s=1\\ \frac{1-H_{2}}{2-H_{1}-H_{2}} & s=2 \end{array}\right.$$
  Note that, $0\leq \omega_{s}\leq 1$, $\overset{n}{\underset{s=1}{\sum }}\omega_{s}=1$.
  Step 4: Integrate the predictions of individual models based on the calculated weights:
  (11) $$\hat{Y}=\sum_{s=1}^{n}\omega_{s}{\hat{y}}_{s}$$
  where ${\hat{y}}_{s}$ is the predictions of the *s*th individual model.
2. Entropy Weight Method B (EWM-B)
  Different from the EWM-A method, the EWM-B method is based on the idea that if an individual prediction model has a smaller entropy value of the prediction error, the variation degree and uncertainty in this model are greater, thereby a smaller weight coefficient should be assigned to this individual model. According to Huang et al. [5], the procedure of integrating the developed improved KNN model and Elman model based on EWM-B are as follows.
  Step 1: Calculate the relative error weight of the individual prediction model:
  (12) $$p_{st}=\frac{\left|e_{st}\right|}{\sum_{t=1}^{m}\left|e_{st}\right|}\;\;(s=1,2,\cdots ,n;t=1,2,\cdots ,m)$$
  where,
  $e_{st}=\left|{\hat{y}}_{st}-y_{t}\right|$,
  *s* indicates different models,
  *n* is the number of individual models (*n* = 2 in this study),
  *t* represents the time,
  *m* is the number of prediction time points,
  ${\hat{y}}_{st}$ is the predicted value of the *s*th individual model at time t,
  $y_{t}$ is the observed value.
  Step 2: Calculate the entropy value of the *s*th individual model:
  (13) $$H_{s}=-k\sum_{t=1}^{m}p_{st}\ln p_{st}\;\;(s=1,2,\cdots ,n)$$
  If $P_{st}=0$, then $P_{st}\ln P_{st}=0$, $k=\frac{1}{\ln m}$
  Step 3: Calculate the variation degree of the *s*th model:
  (14) $$D_{s}=1-H_{s}\;\;\;(s=1,2,\cdots ,n)$$
  where, 0 < *Hs* < 1
  Step 4: Calculate the weight coefficient of the *s*th individual model:
  (15) $$\omega_{s}=\frac{1}{n-1}\left(1-\frac{D_{s}}{\sum_{s=1}^{n}D_{s}}\right)\;\;\;(s=1,2,\cdots ,n)$$
  Note that, in this study *n* = 2, thus:
  (16) $$\omega_{s}=1-\frac{D_{s}}{\sum_{s=1}^{2}D_{s}}=1-\frac{1-H_{s}}{2-H_{1}-H_{2}}=\left\{\begin{array}{cc} \frac{1-H_{2}}{2-H_{1}-H_{2}} & s=1\\ \frac{1-H_{1}}{2-H_{1}-H_{2}} & s=2 \end{array}\right.$$
  Compared with the weight coefficients of EWM-A given in Equation (10), it can be seen that the weight coefficients of two individual models are simply swapped in EWM-B.
  Step 5: Integrate the predictions of individual models based on the calculated weights:
  (17) $$\hat{Y}=\sum_{s=1}^{n}\omega_{s}{\hat{y}}_{s}$$
  where,
  ${\hat{y}}_{s}$ is the predictions of the *s*th individual model.
3. Entropy Weight Method C (EWM-C)
  In information theory, entropy is a measure of the uncertainty associated with a random variable. In the model integration, if we calculate the entropy based on the relative error of the individual prediction model as shown in Equation (7), both low and high accuracy of prediction models could all lead to a small entropy value because the error is relative to other errors. To address this problem, Shan and Zhang [6] proposed to use a new EWM-based method (EWM-C) for model integration to take into account the prediction accuracy levels of the individual models. In this method, they used a weighted entropy of the model prediction error, and the prediction accuracy level of the individual model was incorporated into this weighted entropy. In this way, the impact of the model with low accuracy can be reduced and the prediction accuracy of the integrated model can be improved. Following is the detailed procedure for integrating the prediction models using the EWM-C method.
  Step 1: Calculate the prediction accuracy of the *s*th individual model:
  (18) $$a_{st}=100\%(1-\left|\frac{y_{t}-{\hat{y}}_{st}}{y_{t}}\right|)\;\;\;(s=1,2,\cdots ,n;t=1,2,\cdots ,m)$$
  where,
  $a_{st}$ is the prediction accuracy of the *s*th individual model at time *t*,
  *s* indicates different models,
  *n* is the number of individual models (*n* = 2 in this study),
  *t* represents the time, *m* is the number of prediction time points,
  ${\hat{y}}_{st}$ is the predicted value of the *s*th individual model at time *t*,
  $y_{t}$ is the observed value.
  Step 2: Establish the matrix of model prediction accuracy
  Then, the matrix of the prediction accuracy of different individual models can be expressed as follows:
  (19) $$A_{nm}=\left[\begin{array}{ccc} a_{11} & \cdots & a_{1m}\\ \vdots & \ddots & \vdots \\ a_{n1} & \cdots & a_{nm} \end{array}\right]\;$$
  Note that, the row vector $A_{s}=\left(a_{s1},a_{s2},\;\ldots ,\;a_{sm}\right)$ represents the accuracy of the *s*th individual model $S=\left(1,2,\ldots ,\;n\right)$.
  Step 3: Establish the matrix of accuracy level frequency
  First, round the number in the matrix $A_{nm}$ down to its integer (for example, 87.15% rounded down to 87%). Then, by counting the number of different accuracy levels, the following matrix of the accuracy level frequency can be established.
  (20) $$R_{nm}=\left[\begin{array}{ccc} r_{11} & \cdots & r_{1m}\\ \vdots & \ddots & \vdots \\ r_{n1} & \cdots & r_{nm} \end{array}\right]$$
  where $r_{st}$ represents the number of occurrences of $a_{st}$ (integer part) in the row *s*.
  Step 4: Calculate the weighted information entropy of the *s*th model
  Then, the weighted information entropy of the *s*th model, i.e., *E*_s_, can be calculated by Equation (21).
  (21) $$E_{s}=-\sum_{t=1}^{m}w_{st}p_{st}\log p_{st}\;\;(s=1,2,\cdots ,n)$$
  where,
  (22) $$p_{st}=\frac{r_{st}}{\sum_{t=1}^{m}r_{st}}$$
  (23) $$w_{st}=\left\{\begin{array}{cc} 1 & a_{st}<X\%\\ 1-\frac{N_{st}}{\sum_{t=1}^{m}N_{st}} & a_{st}\geq X\% \end{array}\right\}$$
  *N*_st_ is the number of $a_{st}$ greater than the accuracy level X% in the *s*th row in matrix A (in this study X% = 80%).
  Step 5: Calculate the weight coefficient of the *s*th individual model:
  The weight coefficient of the individual model can be calculated based on the *E_s_* calculated in Step 4 as follows:
  (24) $$\omega_{s}=\frac{1}{ZE_{s}}\;\;(s=1,2,\cdots ,n)\;$$
  where,
  *Z* is a normalization factor that ensures that all weights sum to 1.
  Thus, when *n* = 2, the weight of the two individual models can be calculated as:
  (25) $$\omega_{s}=\left\{\begin{array}{cc} \frac{E_{2}}{E_{1}+E_{2}} & s=1\\ \frac{E_{1}}{E_{1}+E_{2}} & s=2 \end{array}\right.$$
  Step 6: Integrate the predictions of individual models based on the calculated weights:
  (26) $$\hat{Y}=\sum_{s=1}^{n}\omega_{s}{\hat{y}}_{s}$$
  where,
  ${\hat{y}}_{s}$ is the predictions of the *s*th individual model.
  According to the three different EWM-based methods introduced above, different integrated models were developed for each day of the week except Tuesday. The weight coefficients estimated by using different EWM-based methods are presented in Table 1.

## 4. Model Evaluation

**Figure 1:**
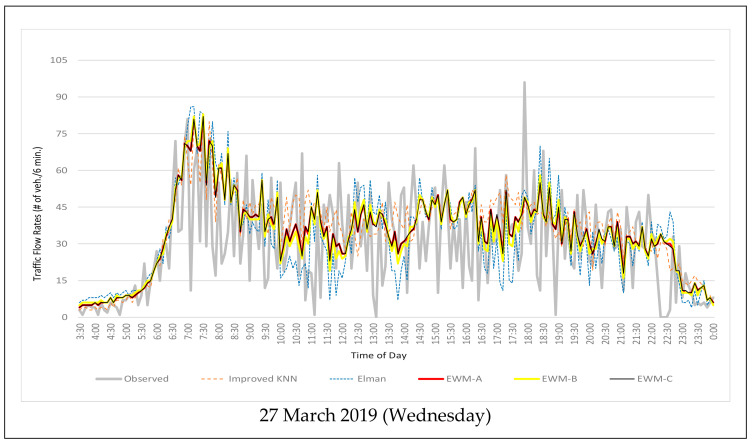

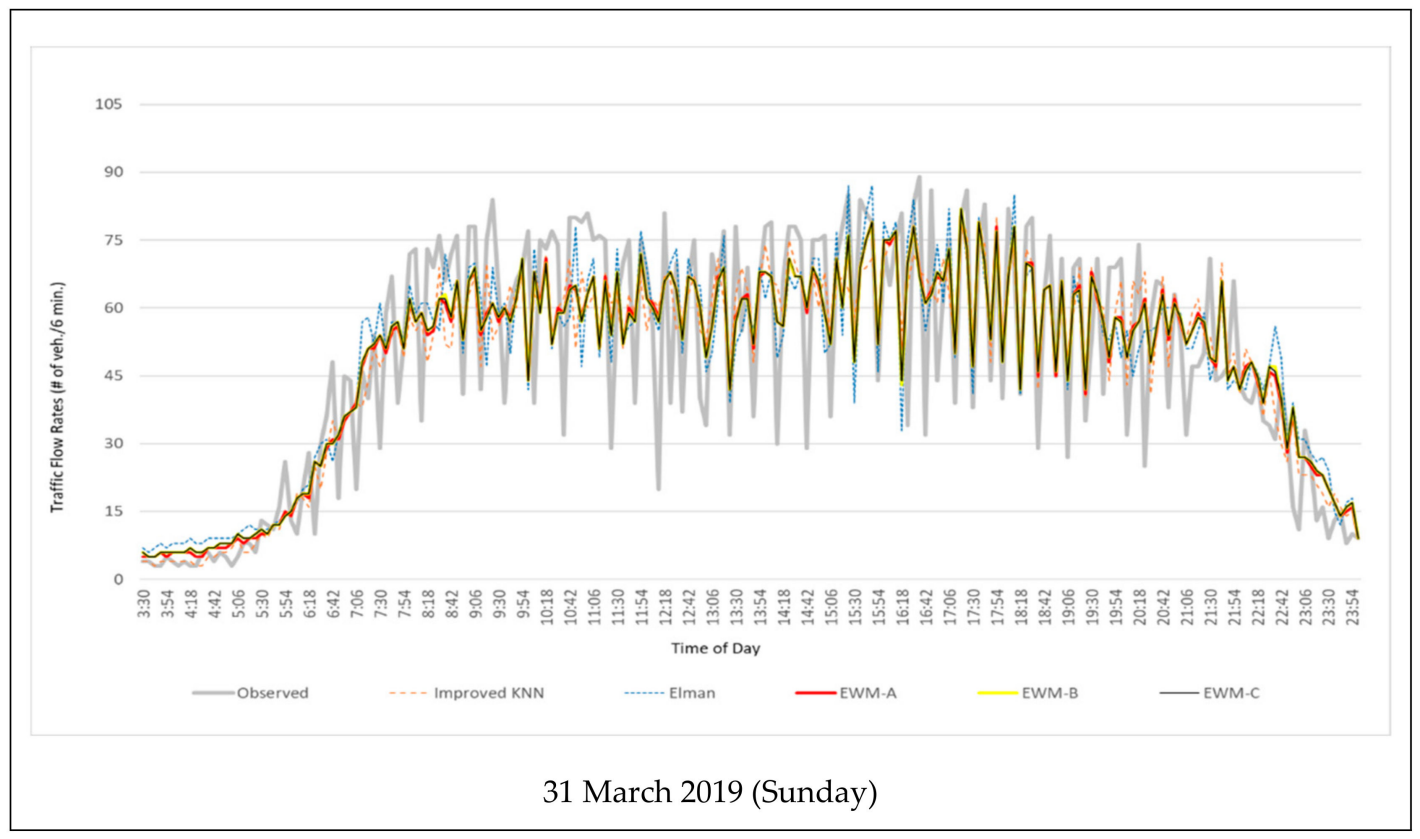
Traffic flow predictions for a weekday and a weekend.

For model evaluation purposes, the developed improved KNN model, Elman model, and the three EWM-based integrated models were applied to the test date, which includes 6 days of traffic flow data collected from 27 March 2019 to 1 April 2019 (Wednesday to Monday). The prediction starts at 3:30 am on each day and after that, a prediction is generated every six minutes. Figure 1 shows the predicted traffic flow rates of different models on 27 March 2019 (Wednesday) and 31 March 2019 (Sunday), along with the observed traffic flow rates on these two days. It can be seen that the traffic flow at this intersection fluctuates more during the weekday. The traffic remains heavy during the weekend while there is an obvious morning peak during the weekdays.

Figure 1 shows that overall the integrated models can predict the trend of traffic flow rate very well. The prediction results of the two individual models have more variance than that of the integrated models. The predicted traffic flow rates of the three integrated models are in the middle of the predicted values of the two individual models. This proves that the integrated model combines the predictions of the improved KNN model and the Elman model.

Next, a performance measure called Mean Square Error (MSE) was used to evaluate the prediction accuracy. MSE measures the differences between the predicted traffic flow rate and observed data and can be calculated as follows:

(27) $$MSE=\frac{\sum_{s=1}^{n}({\hat{y}}_{s}-y_{s}{)}^{2}}{n},$$

where,

${\hat{y}}_{s}\;$represents the predicted traffic flow rate in the *s*th time interval;

$y_{s}\;$represents the observed traffic flow rate in the *s*th time interval;

*n* represents the total number of time intervals in the forecast period.

A smaller MSE value represents a better model performance. MSEs of the models developed for different days are calculated and presented in Table 2. In addition, the results for the three traffic flow prediction models, i.e., Basic BP, KNN, and an integrated model (Elman + KNN) developed in our previous study [21] were included in Table 2 for comparison purposes.

Table 2 shows the improved KNN model outperforms the Elman model on most days. It was also found that the three EWM-based integrated models have better prediction accuracy than the individual models in most cases. This is reasonable because the integrated model can utilize the information provided by both individual models, which leads to improved model prediction accuracy. From Table 2, it can also be seen that, overall, the developed EWM-based integrated models outperform all three models developed in our previous study. In Table 2, we use the bold numbers indicating the best predictions for different days of the week. It is clear that the performance of the integrated model developed using EWM-C is the best on most days and has the lowest average MSE. The accuracy level of the model developed using EWM-A is slightly lower than the one developed using EWM-C. Among the three integrated models, the EWM-B method has the worst performance and it even performs worse than the individual model (improved KNN model) on Wednesday and Thursday (marked in red). The common problem with the EWM-B and EWM-A methods is that the $P_{st}$ in entropy is defined as the ratio of a prediction error to the sum of prediction errors in this model (please see Equation (7)). Thus, if the error in the prediction model increases proportionally, its $P_{st}$ will not change. In other words, the prediction errors $e_{st}$ and $100e_{st}$ will result in the same $P_{st}$ and same weight coefficients, which is unreasonable. In addition, according to the definitions of entropy, the $P_{st}\;$should be a probability instead of a proportion of overall prediction errors. On the other side, in the EWM-C method, the $P_{st}$ is defined as the probability of the prediction error at a given accuracy level (please see Equation (22)). This definition of $P_{st}\;$avoids the problem in EWM-A and EWM-B. In addition, the model accuracy level was directly considered in the weight coefficients given in Equation (23). Thus, more weight will be given to the model with a higher accuracy level, and thereby, the integrated model predictions are more likely to be more accurate than those of the individual models.

## 5. Conclusions and Recommendations

This study investigated the use of the entropy weight method for integrating individual prediction models to improve prediction accuracy. Three different types of entropy weight methods, i.e., EWM-A, EWM-B, and EWM-C, were introduced and applied to develop integrated models for short-term intersection traffic flow prediction. A real-world signalized intersection was selected to collect data for this research. Two individual models, i.e., the improved KNN and Elman models, were developed at first. After that, three integrated models were developed using the three different EWMs. By comparing the performances of the developed models, it was found that the EWM-C model produced more accurate predictions than the other two integrated models. Although EWM-A and EWM-B have been used by many previous studies for model integration purposes, there is a critical problem with the definitions of entropy weight. The entropy should be defined based on the probability of prediction errors instead of the ratio of a prediction error to the sum of prediction errors. This problem will result in unreasonable weight coefficients for the models with different accuracy levels. Thus, both methods, i.e., EWM-A and EWM-B, are not recommended for integrating prediction models. On the other side, in EWM-C, entropy was defined based on the probability of the prediction error at a given accuracy level. This definition avoids the most critical problem in the EWM-A and EWM-B methods and the prediction accuracy level of the individual model was incorporated into the calculated weights. As a result, more weight will be given to the model with a higher accuracy level, which results in improved prediction accuracy. Thus, the EWM-C method was recommended for integrating prediction models.

In this study, we only investigated the three existing EWMs. In the future, more research is needed to investigate how to improve the current EWMs to develop a better EWM for model integration purposes. For example, different thresholds for the model accuracy level in calculating the entropy for EWM-C need to be tested. In addition, the method for integrating more than two models also needs to be investigated. Furthermore, in this study, the traffic data were only collected at one signalized intersection, and due to the lack of traffic flow information on upstream and downstream intersections, the spatial factors cannot be considered in the developed model. In the future, it is necessary to collect more data from more intersections to further refine the developed model.

## Figures and Tables

**Table 1:** KNN model and Elman model weight distribution table.

	Weight	Wed.	Thu.	Fri.	Sat.	Sun.	Mon.
EWM-A	$\omega_{1}$	0.5579	0.5955	0.6189	0.5280	0.5277	0.5599
	$\omega_{2}$	0.4421	0.4045	0.3811	0.4720	0.4723	0.4401
EWM-B	$\omega_{1}$	0.4421	0.4045	0.3811	0.4720	0.4723	0.4401
	$\omega_{2}$	0.5579	0.5955	0.6189	0.5280	0.5277	0.5599
EWM-C	$\omega_{1}$	0.5673	0.5974	0.5738	0.5052	0.4954	0.5602
	$\omega_{2}$	0.4327	0.4026	0.4262	0.4948	0.5046	0.4398

Note: $\omega_{1}$ represent the weights of the improved KNN model. $\omega_{2}\;$ represent the weights of the Elman model.

**Table 2:** Comparison of MSE of different models.

Model	3.27 (Wed.)	3.28 (Thu.)	3.29 (Fri.)	3.30 (Sat.)	3.31 (Sun.)	4.1 (Mon.)	Average
BP * Elman	769.8010 794.0899	544.7767 511.1533	309.5437 262.9230	286.7621 273.4558	212.2913 211.9528	363.5728 319.3155	414.4679 395.4817
KNN * Improved KNN	670.9806 310.5000	534.8592 378.8010	231.0146 226.3252	**243.3155** 298.1456	218.4417 187.6456	284.1707 256.7816	363.7971 276.3665
KNN + Elman *	749.3786	406.6602	251.74272	261.9806	216.8980	274.3980	360.1764
EWM-A EWM-B EWM-C	308.9466	372.3883	208.5777	253.0825	**179.5485**	248.2718	261.8026
	320.8932	398.7524	215.8252	250.0631	181.1650	255.8544	270.4256
	**307.3204**	**371.4563**	**208.4223**	253.0146	180.6845	**248.2718**	**261.5283**

* The models developed by Qu et al. [21].

## Data Availability

The data presented in this study are available on request from the corresponding author. The data are not publicly available due to institutional restrictions.
